# Esmolol Use in Dual Axis Defibrillation Resistant Ventricular Fibrillation

**DOI:** 10.1155/2020/7297303

**Published:** 2020-05-17

**Authors:** Ankit Agrawal, Maria Cardinale, Douglas Frenia, Tarun Dalia, Chirag Shah

**Affiliations:** ^1^Division of Internal Medicine Rutgers Robert Wood, Johnson Medical School/Saint Peter's University Hospital, New Jersey, USA; ^2^Division of Pharmacy, Saint Peter's University Hospital, New Jersey, USA; ^3^Ernest Mario School of Pharmacy, Rutgers University, New Jersey, USA; ^4^Division of Pulmonary and Critical Care Medicine, Rutgers Robert Wood Johnson Medical School/Saint Peter's University Hospital, New Jersey, USA; ^5^Division of Internal Medicine, University of Kansas Medical Center, Kansas, USA; ^6^Division of Cardiovascular Sciences Rutgers, Robert Wood Johnson Medical School/Saint Peter's University Hospital, New Jersey, USA

## Abstract

Cardiac arrest in an event of acute myocardial infarction most commonly results in life-threatening ventricular tachycardia or ventricular fibrillation (VF). Patients who remain in VF despite optimal epinephrine, amiodarone, and three or more attempts at 200 joules of biphasic current defibrillation are known to be in an electrical storm. Here, we describe a case of defibrillation refractory VF responding to intravenous esmolol resulting in a successful return of spontaneous circulation. *Learning objective*. This case reinforces the growing body of evidence supporting esmolol as a novel treatment approach for refractory VF before the cessation of resuscitative efforts.

## 1. Introduction

Sudden cardiac death in cases of acute myocardial infarction commonly results from ventricular tachyarrhythmias such as ventricular tachycardia (VT) or ventricular fibrillation (VF). The development of these malignant arrhythmias may depict ongoing cardiac ischemia. About 18% of in-hospital cardiac arrest (IHCA) present with VF or VT as the initial rhythm [1]. Cardiopulmonary resuscitation (CPR) with guideline-recommended medications like epinephrine and amiodarone and appropriate defibrillation for shockable rhythms remain the cornerstone of advanced cardiac life support (ACLS) [2]. VF is considered refractory if return of spontaneous circulation (ROSC) is not achieved despite at least 10 minutes of high-quality CPR, three defibrillation attempts, 3 mg of epinephrine, and 300 mg of amiodarone [3]. Small observational studies of patients with recurrent VF suggest that beta-blockade may improve long-term survival after cardiac arrest. However, limited data exists supporting the use of intra-arrest beta-blockade. Here, we present a case of defibrillation-refractory VF responding to intravenous intra-arrest esmolol.

## 2. Literature Search

Literature review was done using PubMed and ovid MEDLINE. Keywords like “esmolol”, “refractory ventricular fibrillation”, “double sequential defibrillation”, and “electrical storm” were searched.

## 3. Case

**Figure 1:**
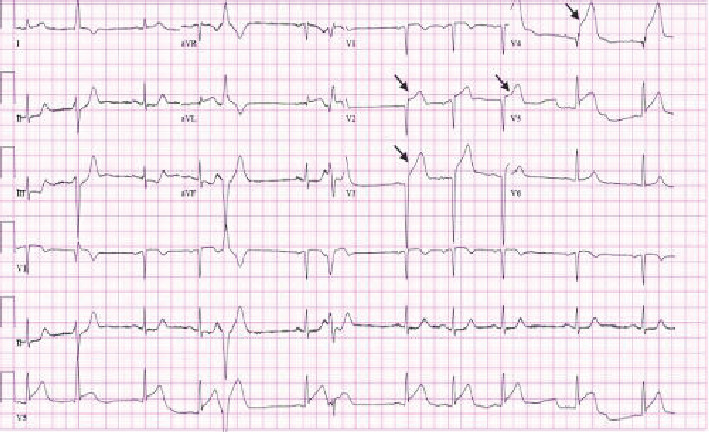
12-lead EKG showing ST segment elevation (black arrows) in leads V2, V3, V4, and V5 suggestive of ST-segment elevation myocardial infarction.

A 52 years old Caucasian male with a history of hypertension presented to the emergency department with severe pressure-like left-sided chest pain of 1-hour duration which radiated to the ipsilateral arm. He was administered 0.4 mg of sublingual nitroglycerin which improved the pain significantly. Vital signs showed a blood pressure of 100/72 mmHg, heart rate of 71/min, respiratory rate of 19/min with a saturation of 96% on room air, and a temperature of 97.8 F. His cardiopulmonary examination was within normal limits. An electrocardiogram was obtained which reflected a new ST segment elevation in leads V2-V5 (Figure 1). The cardiac catheterization laboratory was activated, and the patient was given loading doses of aspirin 325 mg, ticagrelor 180 mg, and atorvastatin 80 mg. Meanwhile, laboratory investigation was significant for troponin-I of more than 80.00 ng/mL. While in the emergency room, the patient started complaining of palpitations and he lost consciousness. Upon examination, there was no palpable pulse and the cardiac monitor showed ventricular fibrillation. A code blue was called and cardiopulmonary resuscitation was initiated. Immediate successful intubation was performed. In total, the patient received 9 rounds of 1 mg intravenous (IV) epinephrine every 3-5 minutes, 300 mg IV amiodarone bolus, 1 gram of 10% calcium chloride, 2 doses of 50 mEq sodium bicarbonate, and 5 biphasic defibrillator shocks of 200 joules (J) each. The cardiac monitor still showed VF.

Considering it as a resuscitation failure, double sequential defibrillation was performed and he received 4 additional shocks of 400 J each. Despite this, the patient was still in an electrical storm and at this moment intravenous esmolol 500 mcg/kg (35 mg) IV bolus was administered. The patient subsequently experienced an immediate return of spontaneous circulation. The total resuscitation time was 50 minutes. The therapeutic hypothermia protocol was initiated, and the patient was urgently transferred to the cardiac catheterization laboratory. A loading dose of tirofiban IV 25 mcg/kg and bivalirudin IV 0.75 mg/kg was administered, and a tirofiban maintenance infusion was continued for 12 hours. Emergent catheterization revealed a 100% occlusion of the left anterior descending artery (LAD) (Video 1) with subsequent placement of two drug-eluting stents (DES) in the LAD (Video 2).

The patient was then promptly transferred to the coronary care unit. Therapeutic hypothermia was stopped after 24 hours and meanwhile echocardiography revealed an ejection fraction of 30% with anterior and apical wall akinesis confirming a diagnosis of ischemic cardiomyopathy. The patient was successfully extubated after 4 days, and his neurological function was at his baseline. His hospital stay was complicated by an isolated episode of atrial fibrillation with rapid ventricular response and aspiration pneumonia. The former responded to beta-blockade for rate control. Aspiration pneumonia was treated with 7 days of intravenous ceftriaxone and metronidazole as per the sensitivity results of sputum culture which grew *Haemophilus influenzae*. The patient was discharged from the hospital on day 12 on aspirin 81 mg once daily, ticagrelor 90 mg two times a day, metoprolol succinate extended-release tablet 150 mg once a day, lisinopril 5 mg once a day, and a life vest. He was followed up within a week at our cardiac rehabilitation clinic where he was found to be asymptomatic and compliant with medications.

## 4. Discussion

The optimal management of VF that is unresponsive to multiple defibrillation attempts is unknown. Several novel interventions have been described in the literature. One such intervention is double sequence defibrillation (DSD). DSD was first described by Hoch and colleagues in 1994 in a case series of five patients in whom DSD was successful in achieving ROSC in VF that was refractory to standard defibrillation [4]. Since then, several case reports demonstrate the potential usefulness of DSD in resuscitation attempts for refractory VF [5]. Extracorporeal cardiopulmonary resuscitation using extracorporeal membrane oxygenation (ECMO) can also be considered in institutions with immediate access to ECMO, as some studies have demonstrated positive results in these patients [6]. Although these strategies show promise, survival rates for refractory VF remain exceptionally poor, and low-quality evidence precludes the widespread adaptation of these strategies into routine ACLS management.

In the present case, DSD was ineffective in terminating the VF arrhythmia, and ECMO was not readily available, so esmolol was administered, with subsequent ROSC. The rationale for esmolol was based on the understanding that beta-adrenergic myocardial hyperstimulation is known to lower the threshold for refractory VF and broaden the ischemic insult in patients with an electrical storm. Electrical storm is defined as the occurrence of three or more episodes of ventricular tachyarrhythmias in 24 hours [7]. Apart from the acute phase of myocardial infarction, an electrical storm can also manifest in the setting of any structural heart disease or genetic arrhythmia syndromes [7]. Activation of the sympathetic nervous system plays a major role in the propagation of electrical storm, and beta-blocking agents are recommended to decrease the incidence of sudden cardiac death [7].

In the setting of cardiac arrest, beta-adrenergic hyperstimulation may be exaggerated as a result of frequent administration of epinephrine in accordance with ACLS guidelines. Esmolol is an intravenous cardioselective beta-1 adrenergic antagonist that is often used in supraventricular tachycardia but also carries an off-label use in the management of electrical storm. In one study of 49 patients with electrical storm and recent myocardial infarction, sympathetic blockade using either beta-blocker agents or left stellate ganglionic blockade was associated with improved survival compared to standard antiarrhythmic therapies [8].

The data supporting the use of beta-blocking therapies during cardiac arrest is very weak. Lee et al. conducted a single-center retrospective prepost study in which sustained ROSC was more common for patients who received esmolol for refractory VF compared to those who did not (*p* value of 0.007) [3]. Esmolol-treated patients were also more likely to be discharged from the hospital with a favorable neurologic outcome (50% vs. 10.5%) [3]. In another retrospective study of 90 cardiac arrest patients with refractory VF performed by Driver et al., patients that received esmolol were more likely to achieve temporary or sustained ROSC, survive to ICU admission, and survive to hospital discharge with good neurological outcomes as compared to those who did not get esmolol [9]. Although these are small, retrospective studies, they demonstrate a potential role for esmolol in refractory VF.

Few case reports describe the successful use of esmolol in refractory VF. Boehm et al. reported a case of refractory VF successfully treated with DSD and esmolol [10]. In this case, DSD was administered at the 15^th^ minute, followed by a bolus of 80 mg of esmolol IV push (approximately 1000 mcg/kg) followed by a continuous infusion. A second DSD was administered, followed by ROSC after 21 minutes of CPR [10]. More recently, Hwang and colleagues described a similar case, in which ROSC was achieved virtually immediately after esmolol was administered [11]. In this case, a dose of 500 mcg of IV esmolol was given, significantly less than the usual bolus dose administered in other tachyarrhythmias of 500 mcg/kg [11]. Like the previous cases, our patient was refractory to both standard defibrillation and DSD. He received five standard defibrillations and four additional DSD in addition to standard ACLS management, with no ROSC. After 45 minutes of CPR, esmolol 500 mcg/kg (35 mg) IV bolus was administered, and the patient achieved ROSC within minutes. In addition, the patient had an excellent neurologic outcome with no apparent sequelae of anoxic brain injury. This case is unique because CPR was performed for a prolonged period of time before ROSC, and he still experienced a favorable outcome. Our patient also received repetitive attempts at DSD without success.

## 5. Conclusion

Refractory VF is a rare but extremely life-threatening condition for which the optimal treatment remains unknown. DSD is a stepping-stone in the management of the same, but cases of shock refractory VF may warrant consideration of the use of beta-adrenergic blockade. This case reinforces the growing body of evidence supporting esmolol as a novel treatment approach for refractory VF before the cessation of resuscitative efforts and describes the successful use of esmolol even in cases of extended CPR time. Prospective studies are needed to define the optimal use of esmolol during cardiac arrest. Until these are available, esmolol may be considered in patients with refractory VF that is unresponsive to standard management.

## Supplementary Materials

Supplementary Materials Video 1: cardiac catheterization revealing complete blockage of the left anterior descending artery with no flow of contrast. Supplementary Materials. Video 2: cardiac catheterization showing the complete restoration of the flow into left anterior descending artery post drug-eluting stent deployment.
